# Supplementary material for: MetaRibo-Seq measures translation in microbiomes
Source: Nat Commun. 2020 Jun 29;11:3268. doi: 10.1038/s41467-020-17081-z (PMC7324362; doi:10.1038/s41467-020-17081-z)
Supplement: Supplementary file 10 — Supplementary Data 7 [file 41467_2020_17081_MOESM10_ESM.zip › File2/Confidence_VeryHigh_Taxonomy/324658_out.krona.html]

Javascript must be enabled to view this page.

members
magnitude
magnitudeUnassigned
count
unassigned
taxon
rank

324658\_out

4

4
superkingdom
2

phylum
1239
4

class
186801
4

order
186802
4

4
family
31979

4
genus
1485

species
1262816

SRS019445\_contig\_number\_32851SRS043001\_contig\_number\_7928SRS147346\_contig\_number\_60153SRS147977\_contig\_number\_7240
4
